# Emergence of Multi-Drug Resistance and Its Association With Uncommon Serotypes of *Streptococcus agalactiae* Isolated From Non-neonatal Patients in Thailand

**DOI:** 10.3389/fmicb.2021.719353

**Published:** 2021-09-08

**Authors:** Orawan Tulyaprawat, Sujiraphong Pharkjaksu, Raj Kumar Shrestha, Popchai Ngamskulrungroj

**Affiliations:** Department of Microbiology, Faculty of Medicine Siriraj Hospital, Mahidol University, Bangkok, Thailand

**Keywords:** drug resistance, serotypes, association, *Streptococcus agalactiae*, non-neonatal patients, multilocus sequence typing, Thailand

## Abstract

Group B streptococcus (GBS) or *Streptococcus agalactiae* is an opportunistic pathogen that causes serious illness in newborns, pregnant women, and adults. However, insufficient detection methods and disease prevention programs have contributed to an increase in the incidence and fatality rates associated with this pathogen in non-neonatal patients. This study aimed to investigate factors of the observed increased incidence by investigation of serotype distribution, virulence factors, and antimicrobial susceptibility patterns from invasive GBS disease among non-neonatal patients in Thailand. During 2017–2018, a total of 109 *S. agalactiae* isolates were collected from non-pregnant patients. There were 62 males and 47 females, with an average age of 63.5 years (range: 20 – 96). Serotypes were determined by latex agglutination assay and multiplex polymerase chain reaction (PCR)-based assay. Among those isolates, seven virulence genes (*rib*, *bca*, *pavA*, *lmb*, *scpB*, *cylE*, and *cfb*) were detected by PCR amplification, and were determined for their susceptibility to 20 antimicrobial agents using a Sensititre^TM^ Streptococcus species STP6F AST plate. Among the study isolates, serotype III was predominant (52.3%), followed by serotype V and serotype VI (13.8% for each), serotype Ib (11.9%), and other serotypes (8.2%). Of the seven virulence genes, *pavA* was found in 67.0%. Except for one, there were no significant differences in virulence genes between serotype III and non-serotype III. Study isolates showed an overall rate of non-susceptibility to penicillin, the first-line antibiotic, of only 0.9%, whereas the resistance rates measured in tetracycline, clindamycin, azithromycin, and erythromycin were 41.3, 22.0, 22.0, and 22.0%, respectively. Strains that were resistant to all four of those drugs were significantly associated with non-serotype III (*p* < 0.001). Using multi-locus sequence typing (MLST), 40.0% of the four-drug-resistant isolates belonged to serotype VI/ST1, followed by serotype Ib/ST1 (35.0%). Cluster analysis with global GBS isolates suggested that the multiple drug-resistant isolates to be strongly associated with the clonal complex (CC) 1 (*p* < 0.001). Compared to the 2014 study of 210 invasive GBS isolates conducted in 12 tertiary hospitals in Thailand, the proportion of serotype III has dramatically dropped from nearly 90% to about 50%. This suggests that resistances to the second-line antibiotics for GBS might be the selective pressure causing the high prevalence of non-serotype III isolates.

## Introduction

Group B streptococcus (GBS) or *Streptococcus agalactiae* is an opportunistic human pathogen that can cause invasive disease in neonates, pregnant women, and non-pregnant adults with chronic medical illness. In healthy adults, GBS often colonizes in the genital and lower gastrointestinal tracts and no symptoms are presented. In newborns, GBS can transmit from the birth canal of colonized mothers during delivery or through aspiration of infected amniotic fluid. The mortality rate among neonates with GBS disease was reported to be as high as 55% (Dermer et al., 2004). In mothers, GBS can be associated with post-partum infections that can lead to maternal bacteremia.

To prevent mortality from neonatal GBS disease, guidelines for diagnosing maternal GBS colonization and prescribing antimicrobial prophylaxis have been introduced, and these guidelines have helped to reduce the mortality rate in neonatal GBS infection from 55% to approximately 5% (Puopolo et al., 2019). Another study reported a 70–90% decrease in the incidence of invasive GBS infection in infants aged 0–6 days (Rao et al., 2017).

Despite the success of the prophylaxis program in neonates, invasive GBS infection has become a serious cause of illness in non-neonatal patients. Due to the lack of a vaccine and prevention and detection strategies in adults, the number of reported GBS infections increased approximately two to four times over the past two decades (Sendi et al., 2008; Lopes et al., 2018), and the mortality rate is estimated to be 15% (High et al., 2005).

The capsular polysaccharide of *S. agalactiae* is used to classify the bacterium into 10 different serotypes (Ia, Ib, and II through IX), and it plays a major role in evading host defense mechanism (Wessels, 1997). Distribution of the capsular serotypes varies geographically and over time. In the 1990s, serotype V was the most frequently reported among GBS isolates from non-pregnant adults in the United States (Blumberg et al., 1996). However, other serotypes have been increasingly reported elsewhere, such as serotype Ia in Portugal (Martins et al., 2012), serotype Ib in Taiwan (Wang et al., 2010) and Japan (Morozumi et al., 2016), serotype III in Hong Kong (Ip et al., 2016), and serotype IV in Canada (Teatero et al., 2015). In contrast, the serotype distribution of invasive GBS among non-neonates in Thailand has yet to be determined.

In addition to the capsular polysaccharide, there are other important factors involved in GBS pathogenesis that influence virulence, such as cell surface antigens, Rib, and alpha antigen of C protein encoded by the *rib* and *bca* genes (Smith et al., 2007; Mudzana et al., 2021). Fibrinogen- and laminin-binding protein for human cell adherence are regulated by *pavA* and *lmb*, respectively (Smith et al., 2007; Tenenbaum et al., 2007). The production of C5a peptidase (*scpB* gene) inhibits neutrophil recruitment (Cheng et al., 2002). In addition, GBS can produce two pore-forming toxins [β-hemolysin (*cylE*) and CAMP factor (*cfb*)] for host-cell lysis leading to several implications in GBS pathogenesis (Pritzlaff et al., 2001; Rosa-Fraile et al., 2014). The detection of potential virulence genes was described in previous studies. The detection of *bca* and *rib* genes identified in GBS strains from the vagina highlighted the potential risk of cervical epithelium invasion and subsequent neonatal meningitis (Souza et al., 2013). Another study reported that neonatal mice infected with *bca* mutation had a five to seven-fold lower mortality rate (Li et al., 1997).

Penicillin is recommended as the first-line antibiotic for treating invasive GBS infection. Although *S. agalactiae* is generally sensitive to penicillin, isolates with reduced susceptibility to penicillin have been reported in Japan since 2008 (Kimura et al., 2008). Another study conducted in Japan reported that the penicillin resistance rate of GBS dramatically increased from 2.3 to 14.7% during 2005–2013 (Hayes et al., 2020). GBS with reduced susceptibility to penicillin has also been reported in Canada (Gaudreau et al., 2010), Korea (Yi et al., 2019), and Germany (van der Linden et al., 2020).

Erythromycin and clindamycin are recommended in penicillin-allergic women or when therapeutic failure is suspected. However, resistance to clindamycin in the United States increased from 37.0 to 43.2% from 2011 to 2016 (Watkins et al., 2019), and half of GBS isolates from non-pregnant adults in the United States were resistant to erythromycin. Increasing rates of resistance to these antibiotics and other drug classes, including macrolide and lincosamide, have also been detected worldwide (Garland et al., 2011; Lopes et al., 2018; Watkins et al., 2019).

Despite of the importance of GBS infections in non-neonatal patients, data specific to GBS in non-neonatal patients in Southeast Asia are scarce. In Thailand, the increased incidence of GBS infections among adults was found from 2013 to 2017 (Phoompoung et al., 2021) and was associated with the underlying disease such as diabetes mellitus and cancer (Paveenkittiporn et al., 2020). However, the association between serotype and antimicrobial susceptibility has never been investigated. Accordingly, this study aimed to investigate serotype distribution, virulence factors and antimicrobial susceptibility pattern of the GBS infection among non-pregnant/non-neonatal patient in Thailand.

## Materials and Methods

### Study Isolates and Identification

During January 2017 to December 2018, all *S. agalactiae* strains isolated from the blood of non-pregnant patients aged not less than 3 years were collected at the Bacteriology Laboratory at the Department of Microbiology, Faculty of Medicine Siriraj Hospital, a large tertiary teaching hospital in Thailand. Ethics approval was granted by the Siriraj Institutional Review Board (COA number: Si 558/2021). The studied isolates were preliminarily identified based on morphology and Christie-Atkins-Munch-Peterson test. The isolates were then confirmed for species identification by matrix assisted laser desorption ionization-time of flight mass spectrometry (MALDI-TOF MS) (Bruker Daltonics, Bremen, Germany) using direct transfer method in accordance with the manufacturer’s instructions (Schulthess et al., 2014). Briefly, a fresh colony grown on blood agar at 37°C in 5% CO_2_ was directly spotted as a thin film onto a ground steel target plate. The spot was then overlaid with 1 μl of a saturated α-cyano-4-hydroxycinnamic acid (HCCA) matrix solution in 50% acetonitrile and 2.5% trifluoroacetic acid and allowed to air-dry. The target plate was analyzed using a Microflex LT mass spectrometer (Bruker Daltonics). Isolates with an identification score of ≥2.0 were reliably identified at species level.

### Genomic DNA Extraction

Isolates were cultured on blood agar at 37°C in 5% CO_2_ for 16–18 h. A fresh colony was inoculated into 5 ml of brain heart infusion broth (Oxoid, United Kingdom) in a 50 ml conical tube. The tube was shaken at 220 rpm at 37°C for 16–18 h. Next, 1.5 ml cultured broth was transferred into a microcentrifuge tube and centrifuged at 10,000 rpm for 5 min. The cell pellet was suspended in 400 μl TE buffer and 50 μl of 10 mg/ml lysozyme. The mixture was incubated at 37°C for 1 h before adding 70 μl of 10% SDS and 5 μl of 20 mg/ml proteinase K. After incubation at 65°C for 30 min, 100 μl of 5 M NaCl and 100 μl of CTAB/NaCl were added. Subsequently, the mixture was incubated at 65°C for 10 min prior to adding 750 μl of chloroform:isoamyl alcohol (24:1). After centrifugation at 10,000 rpm for 5 min, the supernatant was transferred to a new microcentrifuge tube. To remove RNA, 10 μl of RNase A (10 mg/ml) was added and incubated at 37°C for 1 h. The DNA was then precipitated using 0.7 V isopropanol and let stand 1 h at −20°C. After centrifugation at 10,000 rpm for 15 min, DNA pellet was washed with 70% ethanol and centrifuged at 10,000 rpm for 15 min. DNA pellet was air-dried at room temperature and resuspended in TE buffer pH 8.0 for further use.

### Serotyping by Latex Agglutination Assay and Multiplex PCR-Based Assay

All included isolates were designated into serotypes using latex agglutination assay (ImmuLex^TM^ *Streptococcus* Group B Kit; SSI Diagnostica A/S, Copenhagen, Denmark) according to the manufacturer’s recommendations. First, *S. agalactiae* isolates were cultured on blood agar and incubated at 37°C in 5% CO_2_ for 16–18 h. Next, a single colony was selected and suspended in 10 μl phosphate buffer saline (PBS) pH 7.4 on a reaction card. Ten microliters of latex reagent was then dropped next to the suspension and mixed to observed agglutination within 30 s. Each isolate was tested for all 10 latex agglutination reagents specific for each serotype (Ia, Ib, II-IX) of *S. agalactiae*. Visible agglutination was indicative of a positive test for a particular serotype. If no agglutination was observed, the isolates were further serotyped by multiplex polymerase chain reaction (PCR)-based assay. PCR reaction mixture, thermal cycling, and agarose gel electrophoresis were performed according to the original work (Imperi et al., 2010).

### Virulence Gene Detection

Virulence genes of *S. agalactiae* (*rib*, *bca*, *pavA*, *lmb*, *scpB*, *cylE*, and *cfb*) were amplified using PCR. The primer sequences, product sizes, and annealing temperatures are listed in Supplementary Table 1 (Kaczorek et al., 2017). Amplification reactions were performed using One*Taq*^®^ DNA Polymerase (New England Biolabs, Ipswich, MA, United States) in a T100 thermal cycler (Bio-Rad Laboratories, Hercules, CA, United States). With positive and negative control, the PCR amplification protocol was performed, as follows: 30 cycles of denaturation at 94°C for 30 s, annealing depending on each primer for 1 min, and extension at 68°C for 60 s; followed by a final extension at 68°C for 10 min. After amplification, the PCR product was detected by gel electrophoresis. About 10% of positive PCR products from each gene were randomly selected for direct sequencing by Macrogen (Seoul, South Korea). Confirmation of the nucleotide sequences was performed using the Basic Local Alignment Search Tool (BLAST)^1^ [National Center for Biotechnology Information (NCBI), Bethesda MD, United States]. The results showed 98–99% similarity with each specific target (data not shown).

### Antibacterial Susceptibility Testing

The susceptibility of *S. agalactiae* to 20 antimicrobial agents was determined using a Sensititre^TM^ Streptococcus species STP6F AST plate (Thermo Fisher Scientific, Waltham, MA, United States), which is a colorimetric microdilution method, according to the manufacturer’s instructions. First, 3–5 bacterial colonies were suspended in sterile distilled water to create a 0.5 McFarland suspension. Next, 100 μl of the bacterial suspension was transferred into 5 ml of Sensititre^TM^ Mueller Hinton Broth with Lysed Horse Blood to obtain inoculum. One hundred microliters of the inoculum was then inoculated into each well of a Sensititre^TM^ Streptococcus species STP6F susceptibility plate. After 24 h of incubation at 35°C in a non-CO_2_ incubator, the minimum inhibitory concentration (MIC) was determined from the change in the colorimetric growth indicator according to the manufacturer’s instructions. *Streptococcus pneumoniae* (49619; ATCC, Manassas, VA, United States) was used as a quality control. The concentrations of each drug range and interpretive criteria following the instruction of Clinical and Laboratory Standards Institute documents M100S (Wayne et al., 2011) were described in Table 1. MIC range, MIC50, and MIC90 were also reported to show the distribution of AST results. The MIC50 and MIC90 were calculated by the MIC value at which ≥50 and ≥90% of the isolates are inhibited, respectively (Schwarz et al., 2010).

**TABLE 1:** Susceptibility to 20 antimicrobial agents, minimal inhibitory concentrations (MICs), and serotype group of *Streptococcus agalactiae* strains.

**Antimicrobial agents**	**Dilution range (μg/ml)**	**CBPs (μg/ml)**			**MIC (μg/ml)**			**Number (%) of resistant/non-susceptible isolates**			
		**S**	**I**	**R**	**MIC range**	**MIC50**	**MIC90**	**All**	**Serotype III**	**Non-serotype III**	***P*-value**
Tetracycline	1–8	≤2	4	≥8	≤1 to >8	≤1	>8	45 (41.3%)	5 (8.8%)	40 (76.9%)	***<0.001***
Clindamycin	0.12–1	≤0.25	0.5	≥1	≤0.12 to >1	≤0.12	>1	24 (22.0%)	4 (7.0%)	20 (38.5%)	***<0.001***
Erythromycin	0.25–2	≤0.25	0.5	≥1	≤0.25 to >2	≤0.25	>2	24 (22.0%)	6 (10.5%)	18 (34.6%)	***0.002***
Azithromycin	0.25–2	≤0.5	1	≥2	≤0.25 to >2	≤0.25	>2	24 (22.0%)	6 (10.5%)	18 (34.6%)	***0.005***
Chloramphenicol	1–32	≤4	8	≥16	2–32	4	4	2 (1.8%)	0 (0.0%)	2 (3.8%)	NA
Levofloxacin	0.5–4	≤2	4	>4	≤0.5 to >4	1	1	2 (1.8%)	0 (0.0%)	2 (3.8%)	NA
Penicillin	0.03–4	≤0.12	–	–	≤0.03–0.25	0.06	0.06	1 (0.9%)	0 (0.0%)	1 (1.9%)	NA
Vancomycin	0.5–4	≤1	–	–	≤0.5–1	≤0.5	≤0.5	0 (0.0%)	0 (0.0%)	0 (0.0%)	NA
Daptomycin	0.06–2	≤1	–	–	0.5–1	0.5	0.5	0 (0.0%)	0 (0.0%)	0 (0.0%)	NA
Meropenem	0.25–2	≤0.5	–	–	≤0.25–0.5	≤0.25	≤0.25	0 (0.0%)	0 (0.0%)	0 (0.0%)	NA
Ertapenem	0.5–4	≤0.5	–	–	≤0.5	≤0.5	≤0.5	0 (0.0%)	0 (0.0%)	0 (0.0%)	NA
Ceftriaxone	0.12–2	≤0.5	–	–	≤0.12	≤0.12	≤0.12	0 (0.0%)	0 (0.0%)	0 (0.0%)	NA
Cefotaxime	0.12–4	≤0.5	–	–	≤0.12	≤0.12	≤0.12	0 (0.0%)	0 (0.0%)	0 (0.0%)	NA
Cefepime	0.5–8	≤0.5	–	–	≤0.5	≤0.5	≤0.5	0 (0.0%)	0 (0.0%)	0 (0.0%)	NA
Linezolid	0.25–4	≤2	–	–	1–2	1	2	0 (0.0%)	0 (0.0%)	0 (0.0%)	NA
Amoxicillin/clavulanic acid 2:1 ratio	2/1–16/8	NA	NA	NA	≤2/1	≤2/1	≤2/1	NA	NA	NA	NA
Cefuroxime	0.5–4	NA	NA	NA	≤0.5	≤0.5	≤0.5	NA	NA	NA	NA
Moxifloxacin	1–8	NA	NA	NA	≤1	≤1	≤1	NA	NA	NA	NA
Tigecycline	0.015–0.12	NA	NA	NA	≤0.015–0.12	0.06	0.06	NA	NA	NA	NA
Trimethoprim/sulfamethoxazole	0.5/9.5–4/76	NA	NA	NA	≤0.5/9.5	≤0.5/9.5	≤0.5/9.5	NA	NA	NA	NA
Total								109 (100%)	57 (100%)	52 (100%)	

*A *P*-value < 0.05 indicates statistical significance (chi-square test), highlighted by a bold and italic number.*

*CBPs, clinical breakpoints; MIC, minimum inhibitory concentration; S, susceptible; I, intermediate; R, resistant; NA, not available.*

### Multi-Locus Sequence Typing (MLST) and MLST-Based Phylogenetic Tree Analysis

All 20 isolates resistant to all 4 drugs including tetracycline, clindamycin, azithromycin, and erythromycin were analyzed genetic relationship with 11 selected susceptible isolates (4 serotype III; 2 serotype IV; 2 serotype V, 2 serotype VI; and, 1 serotype Ib) by using MLST-based dendrogram. First, seven housekeeping genes of *S. agalactiae* (*adhP*, *pheS*, *atr*, *glnA*, *sdhA*, *glcK*, and *tkt*) were amplified, as described previously (Jones et al., 2003). The PCR products were purified and sequenced in both forward and reverse directions by Macrogen (Seoul, South Korea). The retrieved nucleotide sequences were edited by MEGAX software.^2^ Subsequently, the edited sequences were entered in the *S. agalactiae* database^3^ to assign allele numbers (ATs) and identify sequence types (STs). After that, the seven loci were concatenated to construct the phylogenetic tree using the unweighted pair-group method with arithmetic mean (UPGMA) method. To compare the relationship between the resistant isolates and *S. agalactiae* population worldwide, isolates that had been deposited in the MLST database as of August 2021 were obtained to construct a phylogenetic tree. Subsequently, a minimum spanning tree was constructed to illustrate relativeness between genetic lineage and antibiotic-resistant patterns using Bionumerics software (Applied Maths, Belgium). Evolutionary lineage among isolates was defined as a clonal complex (CC) using the goeBURST algorithm.^4^

### Statistical Analysis

Association analysis was performed using PASW Statistics for Windows (SPSS Inc., Chicago, IL, United States). Using a chi-square test, a *P*-value < 0.05 was considered statistically significant.

## Results

### Serotype Distribution of Blood Isolates From Non-neonatal Thai Patients

From 2017 to 2018, a total of 109 *S. agalactiae* isolates were collected from non-pregnant patients. There were 62 males and 47 females, with an average age of 63.5 ± 16.7 (1 SD) years old (ranging from 20 to 96 years old). The majority of isolates were serotype III (52.3%, 57/109 isolates). The serotype V and serotype VI ranked as the second most common (13.8%, 15/109 isolates for each serotype), followed by serotype Ib (11.9%, 13/109 isolates). Other isolates were serotype Ia (3.7%, 4/109 isolates), serotype VII (2.7%, 3/109 isolates), and serotype IV (1.8%, 2/109 isolates). No serotype II, VIII, or IX were found.

### Virulence Gene Profiling

The seven most common virulence genes detected in *S. agalactiae* were selected for analysis (Kaczorek et al., 2017). The proportions of positive detection are shown in Table 2. Among the detected genes, *pavA* was the most frequently found at 67.0%. Since serotype III was much more common than the other serotypes, study isolates were divided into two groups for comparison. Isolates belonging to serotype III were classified as a common serotype, whereas isolates belonging to any other serotypes were defined as uncommon serotypes. However, the difference in the *pavA* positive rate between serotype III and non-serotype III was not statistically significant. In contrast, the positive rate of *scpB* in the non-serotype III group was significantly higher than that in the serotype III group (48.1 vs. 26.3%, respectively).

**TABLE 2:** Rates of positive detection of the seven virulence genes among *Streptococcus agalactiae* strains for all isolates, and compared between serotype III and non-serotype III.

**Target gene**	**Number (%) of positive detection isolates**			***P***-**value**
	**All**	**Serotype III**	**Non-serotype III**	
*pavA*	73(67.0%)	38(66.7%)	35(67.3%)	0.943
*cfb*	70(64.2%)	33(57.9%)	37(71.2%)	0.149
*lmb*	60(55.0%)	34(59.6%)	26(50.0%)	0.312
*cylE*	49(45.0%)	26(45.6%)	23(44.2%)	0.885
*bca*	47(43.1%)	25(43.9%)	22(42.3%)	0.870
*scpB*	40(36.7%)	15(26.3%)	25(48.1%)	***0.019***
*rib*	39(35.8%)	23(40.4%)	16(30.8%)	0.297
Total	109(100%)	57(100%)	52(100%)	

*A *P*-value < 0.05 indicates statistical significance (chi-square test), highlighted by a bold and italic number.*

### Antimicrobial Susceptibility Profiles

The results of antimicrobial susceptibility testing to 20 antimicrobial agents are shown in Table 1. The rate of non-susceptibility to penicillin was only 0.9%. The MIC90 of penicillin determined in this study was 0.06 μg/ml, whereas only one resistant isolate showed the highest MIC of 0.25 μg/ml. Similarly, only two isolates each were resistant to levofloxacin and chloramphenicol (1.8% for each). In contrast, the rates of resistance to tetracycline, clindamycin, azithromycin, and erythromycin were 41.3, 22.0, 22.0, and 22.0%, respectively.

Among the resistant isolates, 18.3% (20/109 isolates) were multi-drug resistant to tetracycline, clindamycin, erythromycin, and azithromycin. The multi-drug resistant isolates belonged to serotype Ib (45.0%), serotype VI (40.0%), and serotype III (15.0%) (Supplementary Table 2). Comparison between serotype and antimicrobial susceptibility showed that the proportions of resistance to tetracycline, clindamycin, erythromycin, and azithromycin among non-serotype III isolates was significantly higher than those among the serotype III isolates (Table 1).

### Multi-Locus Sequence Typing-Based Phylogenetic Tree and Multiple Drug Resistance

**FIGURE 1:**
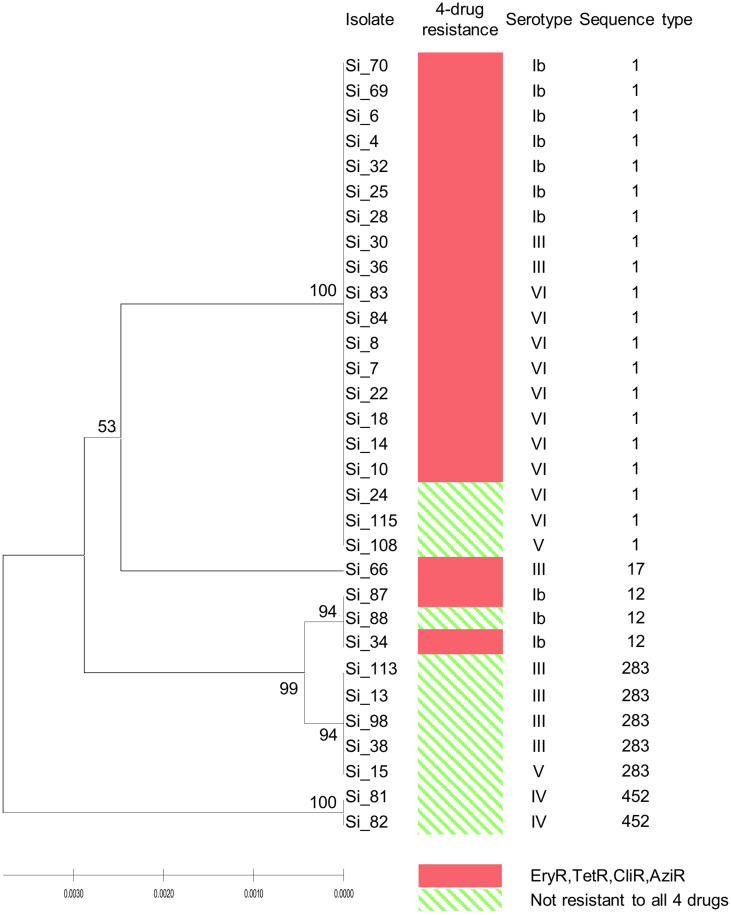
UPGMA-based dendrogram of 7 housekeeping genes of 20 isolates resistant to tetracycline, clindamycin, azithromycin, and erythromycin, and 11 susceptible isolates from this study. Ery, erythromycin; Tet, tetracycline; Cli, clindamycin; Azi, azithromycin; R, resistant.

**FIGURE 2:**
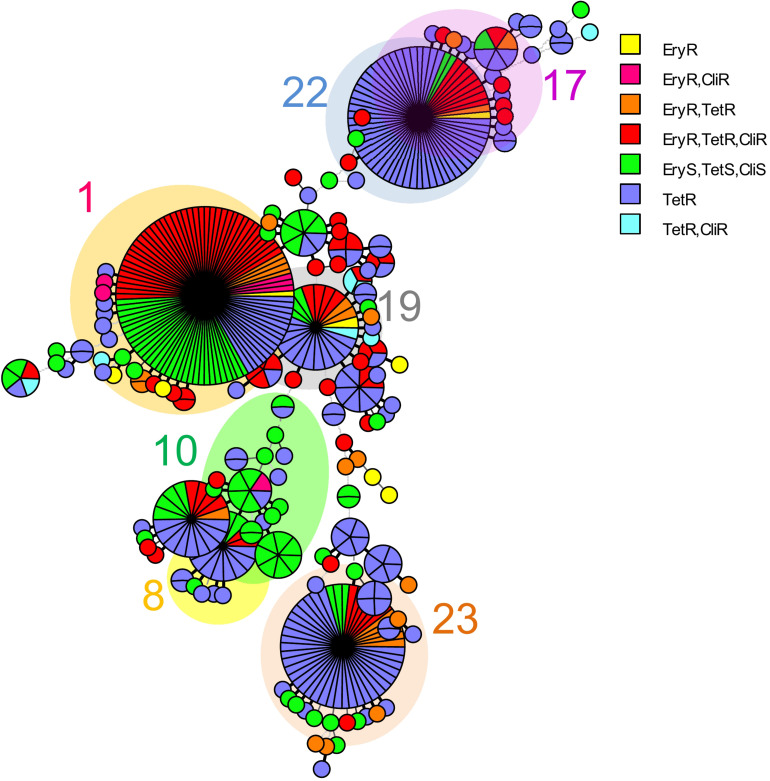
A minimum spanning tree illustrated the relationship between the 31 *S. agalactiae* isolates from Thailand and 446 isolates with available susceptibilities of tetracycline, clindamycin, and erythromycin from the *S. agalactiae* MLST database as of August 2021. Each circle corresponds to a unique ST; the number outside the circle indicates a clonal complex; the size of the circle represents the number of isolates belonging to the same ST; and the colors inside the circle represent tetracycline, clindamycin, and erythromycin susceptibility. Tet, tetracycline; Cli, clindamycin and Ery, erythromycin.

Analysis of MLST revealed that most of the four drugs resistant isolates belonged to serotype VI/ST1 (40.0%) and serotype Ib/ST1 (35.0%). The others belonged to in serotype III/ST1 (10.0%), serotype Ib/ST12 (10.0%), and serotype III/ST17 (5.0%). The UPGMA-based dendrogram demonstrated that resistance to the four drugs was not assigned to a particular clade, as illustrated in Figure 1. However, this might have a limitation on a few resistant patterns and isolate numbers in the present study. Therefore, we further compared our isolates to global GBS isolates that had susceptibilities of tetracycline, clindamycin, and erythromycin. A total of 446 GBS isolates with different 150 STs was retrieved from the pubMLST database for categorical analysis. A minimum spanning tree illustrated that the three drugs resistant isolates found in several CCs and overrepresented in CC1 (*p* < 0.001; Figure 2 and Table 3). Approximately 50% isolates in the CC1 belonged to serotype Ib/ST1 and serotype VI/ST1 (Supplementary Figure 1).

**TABLE 3:** Distribution of MLST-based clonal complex (CC) among erythromycin, tetracycline, and clindamycin resistance phenotypes of the 31 *S. agalactiae* isolates from Thailand and 446 global isolates.

**Clonal complex**	**Total number (%)**	**Number of isolates among Ery, Tet, Cli resistance patterns (%)**							***P*-value^b^**
		**EryS, TetS, CliS**	**EryR**	**TetR**	**EryR, TetR**	**EryR, CliR**	**TetR, CliR**	**EryR, TetR, CliR**	
CC 1	118 (24.7)	34 (34.7)	3 (37.5)	26 (11.6)	6 (22.2)	5 (83.3)	0 (0.0)	44 (41.9)	***<0.001***
CC17	77 (16.1)	3 (3.1)	1 (12.5)	59 (26.2)	2 (7.4)	0 (0.0)	0 (0.0)	12 (11.4)	0.137
CC 23	59 (12.4)	7 (7.1)	0 (0.0)	38 (16.9)	7 (25.9)	0 (0.0)	0 (0.0)	7 (6.7)	***0.044***
CC 19	58 (12.2)	8 (8.2)	2 (25.0)	23 (10.2)	4 (14.8)	0 (0.0)	6 (75.0)	15 (14.3)	*0.450*
CC 8	25 (5.2)	5 (5.1)	0 (0.0)	17 (7.6)	0 (0.0)	0 (0.0)	0 (0.0)	3 (2.9)	*0.215*
CC 12	22 (4.6)	5 (5.1)	0 (0.0)	11 (4.9)	1 (3.7)	0 (0.0)	0 (0.0)	5 (4.8)	*0.934*
CC 10	19 (4.0)	14 (14.3)	0 (0.0)	3 (1.3)	0 (0.0)	1 (16.7)	0 (0.0)	1 (1.0)	*0.072*
Others^a^	99 (20.8)	22 (22.4)	2 (25.0)	48 (21.3)	7 (25.9)	0 (0.0)	2 (25.0)	18 (17.0)	*0.301*
Total	477 (100.0)	98 (100.0)	8 (100.0)	225 (100.0)	27 (100.0)	6 (100.0)	8 (100.0)	105 (100.0)	

*^*a*^Other clonal complexes including CC 2, CC 4, CC 22, CC 24, CC 28, CC88, CC 144, CC196, CC 297, CC 485, and singletons.*

*^*b*^A *P*-value < 0.05 indicates statistical significance (chi-square test) of association between EryR, TetR, CliR, and clonal complexes, highlighted by a bold and italic number.*

*CC, clonal complex; Tet, tetracycline; Cli, clindamycin; Ery, erythromycin; S, susceptible; R, resistant.*

## Discussion

In this study, serotype III was the most prevalent serotype (52.3%) of invasive GBS infection among non-neonatal patients. Compared to a previous multi-center study that collected 210 GBS isolates from 12 tertiary hospitals in Thailand, the proportion of serotype III has dramatically dropped from about 87 to 52.3% within 4 years (Kerdsin et al., 2017). Similar to our study, the average age and age range in that study was 57.2 years and 22–99 years, respectively. Unfortunately, the pregnant status of patients was not described in the previous finding. Compared to other countries, the predominance of serotype III among non-neonates in Thailand is in agreement with similar predominance reported from China, Belgium, and France (Tazi et al., 2011; Jiang et al., 2016; Graux et al., 2020). However, the proportion of serotype III isolated in Thailand was higher than the proportions reported from those three counties (China 32.2%, Belgium 21.0%, and France 25.7%). In contrast, serotype V was the most frequently identified serotype in Poland, Norway, Sweden, Australia, and New Zealand (Bergseng et al., 2008; Zhao et al., 2008; Gudjónsdóttir et al., 2015; Kaminska et al., 2020).

The observed increase in the prevalence of non-serotype III was further investigated for a factor related to the mechanism of change in virulence *via* virulence gene detection. However, except for one gene, there was no significant difference in virulence genes between serotype III and non-serotype III. Only *scpB* showed a significantly higher frequency among non-serotype III compared to serotype III. No significant difference was found between groups for *lmb*, which is a functional gene that is closely related to *scpB*. Moreover, our result was inconsistent with that from a previous study conducted in Thailand (Kerdsin et al., 2017). Those authors reported that GBS serotype III from Nile tilapia farms had higher virulence and pathogenesis than the other serotypes. However, the result might vary by host species. The results of our analysis suggest that the increased prevalence of non-serotype III isolates is not related to a change in virulence genes.

We further investigated for changes in the antimicrobial susceptibility profile as a suspected factor of the observed increase in non-serotype III prevalence. Almost all isolates (99.1%) were still susceptible to penicillin, while the resistance rates of tetracycline, clindamycin, azithromycin, and erythromycin were high. This result suggests that a substantial increase in the prevalence of non-serotype III isolates, which were uncommon serotypes in Thailand, might be associated with an emergence of multiple drug resistance. Since most antibiotics are available over-the-counter and without prescription in Thailand (Sommanustweechai et al., 2018; Chanvatik et al., 2019), the susceptible serotype III, which is in the gastrointestinal and genitourinary tract microbiota of most adults, might be activated by overuse of these antibiotics. As a result, the more drug-resistant non-serotype III may become more prevalent than the drug-susceptible serotypes.

Previous studies reported that GBS serotype III highly correlated with levofloxacin and erythromycin resistance (Jiang et al., 2016; Kao et al., 2019), but the same correlation was not found in the present study. Our finding of non-serotype III being associated with tetracycline, clindamycin, azithromycin, and erythromycin resistance may be due to differences in genetic lineages. Therefore, MLST was employed to determine the STs and evolutionary lineages of the isolates. Jiang et al. (2016) and Kao et al. (2019) found serotype III/ST19 and serotype III/ST17 to be associated with levofloxacin and erythromycin resistance, respectively; however, the serotype III/ST17 was found only 1 of the 20 resistant isolates. This might be caused by geographic variation. Consequently, the same correlation was not found in this study.

On the other hand, serotype Ib/ST1 and serotype VI/ST1 were most frequently found to have second-line antibiotics resistance. Furthermore, an overrepresentation of the multi-drug resistance was found in CC1. The correlation between CC1 and macrolide resistance was previously found among neonates GBS in Portugal (Martins et al., 2017). In addition, dissemination of invasive serotype VI/ST1 belonging to CC1 was reported in central Taiwan in 2016 (Lin et al., 2016). However, the number of GBS isolates was limited and the number of the non-serotype III associated second-line antibiotic-resistant isolates was relatively small in our study. Further investigation including multiple centers from intra- and inter-laboratory will be needed for a better understanding of epidemiology and its relationship to the drugs resistance of invasive GBS circulating in Thailand.

## Conclusion

Compared to the results of a 2014 study conducted in Thailand, the proportion of serotype III has dramatically dropped from nearly 90% to about 50%. This finding suggests that second-line antibiotic resistance might be the selective pressure causing the high prevalence of non-serotype III isolates, and this likely contributes to the high mortality rate among non-neonates with GBS infection. Screening of GBS in the gastrointestinal microbiota, monitoring of serotype distribution and antibiotic resistance from intra- and inter-laboratory studies are needed.

## Data Availability Statement

The original contributions presented in the study are included in the article/Supplementary Material, further inquiries can be directed to the corresponding author.

## Ethics Statement

The studies involving human participants were reviewed and approved by the Siriraj Institutional Review Board. Written informed consent for participation was not required for this study in accordance with the national legislation and the institutional requirements.

## Author Contributions

PN contributed to the conception and design of this study and assembled the final version of the manuscript. RS recovered the bacterial isolates and provided the DNA. OT identified the bacterial species and serotyped and conducted the majority of data analysis. SP was responsible for the virulence gene detection. OT and SP performed the antimicrobial susceptibility and MLST analysis. OT, SP, and RS worked under the supervision of PN. All authors contributed to manuscript revision and approved the submitted version.

## Conflict of Interest

The authors declare that the research was conducted in the absence of any commercial or financial relationships that could be construed as a potential conflict of interest.

## Publisher’s Note

All claims expressed in this article are solely those of the authors and do not necessarily represent those of their affiliated organizations, or those of the publisher, the editors and the reviewers. Any product that may be evaluated in this article, or claim that may be made by its manufacturer, is not guaranteed or endorsed by the publisher.

## Footnotes

1
http://blast.ncbi.nlm.nih.gov/Blast.cgi

2
https://www.megasoftware.net/

3
https://pubmlst.org/organisms/streptococcus-agalactiae/

4
https://phyloviz.readthedocs.io/

## Supplementary Material

Supplementary Figure 1 A minimum spanning tree showing serotype/sequence type related to a clonal complex of the 31 *S. agalactiae* isolates from Thailand and 446 isolates with available susceptibilities of tetracycline, clindamycin, and erythromycin from the *S. agalactiae* MLST database as of August 2021. Each circle corresponds to a unique ST; the number outside the circle indicates a clonal complex; the size of the circle represents the number of isolates belonging to the same ST; and the colors inside the circle represent each serotype/sequence type.

Supplementary Table 1 The primer sets used for detection of virulence genes in this study (Kaczorek et al., 2017). bp, base pair.

Supplementary Table 2 Results of serotype, virulent gene detection, and antimicrobial susceptibility of 109 *S. agalactiae* isolates in this study.
