# Supplementary material for: Emergence of Multi-Drug Resistance and Its Association With Uncommon Serotypes of Streptococcus agalactiae Isolated From Non-neonatal Patients in Thailand
Source: Front Microbiol. 2021 Sep 8;12:719353. doi: 10.3389/fmicb.2021.719353 (PMC8456118; doi:10.3389/fmicb.2021.719353)

**Supplementary Table 1.** The primer sets used for detection of virulence genes in this study (Kaczorek, *et al*., 2017)

| **Target gene** | **Primer sequence (5′–3′)** | **Amplicon**  **size (bp)** | **Annealing**  **temperature (°C)** |
| --- | --- | --- | --- |
| *rib* | F: CAGGAAGTGCTGTTACGTTAAAC  R: CGTCCCATTTAGGGTCTTCC | 369 | 51°C |
| *bca* | F: TAACAGTTATGATACTTCACAGAC  R: ACGACTTTCTTCCGTCCACTTAGG | 535 | 49°C |
| *pavA* | F: TTCCCATGATTTCAACAACAAG  R: AACCTTTTGACCATGAATTGGTA | 495 | 47°C |
| *lmb* | F: AGTCAGCAAACCCCAAACAG  R: GCTTCCTCACCAGCTAAAACG | 397 | 50°C |
| *scpB* | F: AGTTGCTTCTTACAGCCCAGA  R: GGCGCAGACATACTAGTTCCA | 567 | 51°C |
| *cylE* | F: TGACATTTACAAGTGACGAAG  R: TTGCCAGGAGGAGAATAGGA | 248 | 47°C |
| *cfb* | F: ATGGGATTTGGGATAACTAAGCTAG  R: AGCGTGTATTCCAGATTTCCTTAT | 193 | 50°C |

**Abbreviation:** bp, base pair

**Supplementary Figure 1.** A minimum spanning tree showing serotype/sequence type related to a clonal complex of the 31 *S. agalactiae* isolates from Thailand and 446 isolates with available susceptibilities of tetracycline, clindamycin and erythromycin from the *S. agalactiae* MLST database as of August 2021. Each circle corresponds to a unique ST; the number outside the circle indicates a clonal complex; the size of the circle represents the number of isolates belonging to the same ST; and the colors inside the circle represent each serotype/sequence type.


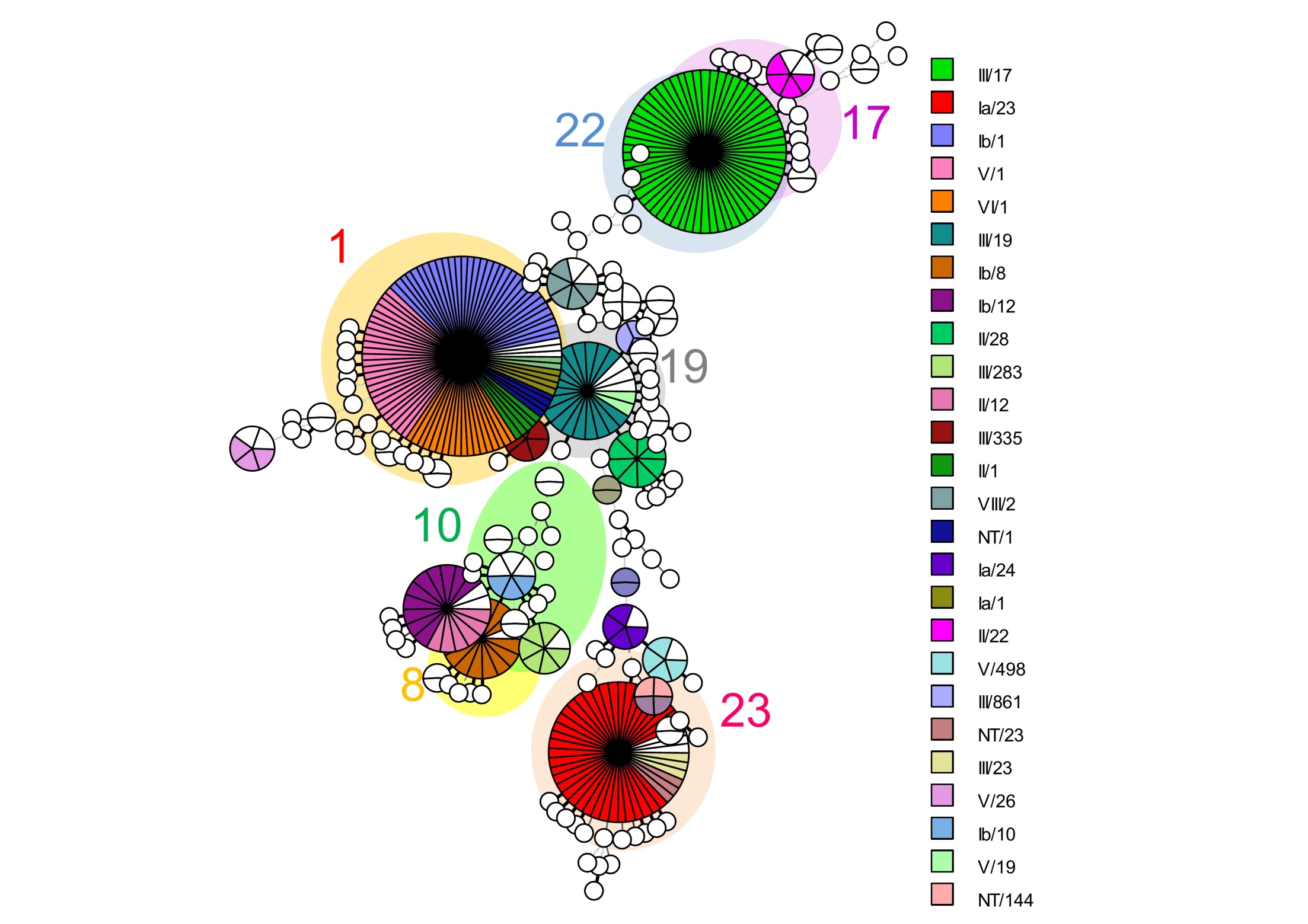

Supplement: Supplementary Figure 1 — A minimum spanning tree showing serotype/sequence type related to a clonal complex of the 31 S. agalactiae isolates from Thailand and 446 isolates with available susceptibilities of tetracycline, clindamycin, and erythromycin from the S. agalactiae MLST database as of August 2021. Each circle corresponds to a unique ST; the number outside the circle indicates a clonal complex; the size of the circle represents the number of isolates belonging to the same ST; and the colors inside the circle represent each serotype/sequence type. [file Data_Sheet_1.docx]
